# Edema Surrounding Benign Tumors and Tumor-Like Lesions

**DOI:** 10.1155/2019/8206913

**Published:** 2019-10-29

**Authors:** Sai Gao, Ruizhi Zhou, Qi Xu, Haisong Chen

**Affiliations:** Department of Radiology, The Affiliated Hospital of Qingdao University, Qingdao 266003, China

## Abstract

**Objective:**

To explore the incidence and significance of intra- and extra-osseous edema associated with benign tumors and tumor-like diseases.

**Methods:**

Magnetic resonance imaging (MRI) data from 300 benign osseous tumors and tumor-like diseases diagnosed by pathology were retrospectively reviewed. Borderline tumors, cases associated with pathological fractures, and skull lesions were excluded from the study. Bone marrow and soft tissue edema were defined on T2WI with fat suppression on MRI in all cases. The incidence rate of edema in benign tumors and tumor-like diseases was determined using the *χ*^2^ test. The preoperative diagnoses were reviewed, and the effect of edema on the differential diagnosis of benign and malignant tumors was analyzed.

**Results:**

The incidence rate of bone marrow and soft tissue edema associated with benign tumors and tumor-like diseases was 35.7% (107/300), including 84.4% (27/32) Langerhans cell histiocytosis, 86.4% (19/22) osteoblastoma, 93.9% (31/33) osteoid osteoma, and 85.2% (23/27) chondroblastoma cases. There was no statistically significant difference in the incidence of edema among the four diseases (*χ*^2^ = 1.7, *P* > 0.05). Of 107 cases associated with edema, 49 (45.8%) were misdiagnosed as malignant tumors by MRI preoperatively.

**Conclusion:**

Bone marrow and soft tissue edema are a common finding associated with benign bone tumors and tumor-like diseases, and they are frequently detected in Langerhans cell histiocytosis, osteoblastoma, osteoid osteoma, and chondroblastoma.

## 1. Introduction

The incidence of bone tumors and tumor-like lesions is 2%–3%, and the ratio of benign to malignant tumors is 0.83 : 1 [1]. The incidence of benign tumors and tumor-like lesions is 0.91%–1.36%; however, the incidence may be underestimated because benign lesions are usually asymptomatic and frequently remain undiscovered [2]. Benign tumors and tumor-like lesions are more likely to occur in patients aged 11–30 years with a ratio of men to women of 1.5 : 1, and the most common locations are the femur and tibia. The treatment of benign bone tumors and tumor-like lesions includes surgery, chemotherapy, radiotherapy, and interventional therapy. Asymptomatic benign tumors and tumor-like lesions can be monitored as a follow-up [3].

Bone marrow and soft tissue edema are a common finding associated with benign and malignant tumors or tumor-like diseases. In routine clinical practice, a lesion that is associated with remarkable surrounding edema is considered as an invasive active tumor by radiologists [4–6]. Yamamura et al. [7] showed that obvious surrounding edema disappeared and high serum levels of prostaglandin decreased after the resection of lesions in patients with osteoid osteoma and osteoblastoma. This phenomenon suggests that the inflammatory medium may lead to the development of soft tissue and marrow edema. The destruction of trabeculae increased pressure inside the bone marrow, discharging of inflammatory medium, and liquid exudate from capillary vessels inside tumors were suggested as possible causes of marrow and soft tissue edema surrounding a lesion [8, 9]. Hence, differentiating benign from malignant lesions with surrounding edema is important to prevent unnecessary surgery or over-resection of lesions with a larger area [10]. We retrospectively reviewed pathologically proven benign bone tumors and tumor-like diseases to explore the occurrence rate and characteristics of edema surrounding these lesions.

The diagnosis of bone marrow and soft tissue edema was based on the following criteria: magnetic resonance imaging (MRI) was used because it is sensitive for the detection of edema [5]. An abnormal signal, such as long T1 and long T2 compared with the surrounding normal bone marrow or soft tissue, and a high signal on T2WI with fat-suppression or a STIR sequence with a vague margin were considered indicators of bone marrow or soft tissue edema [6].

## 2. Materials and Methods

### 2.1. Patients

**Figure 1:**
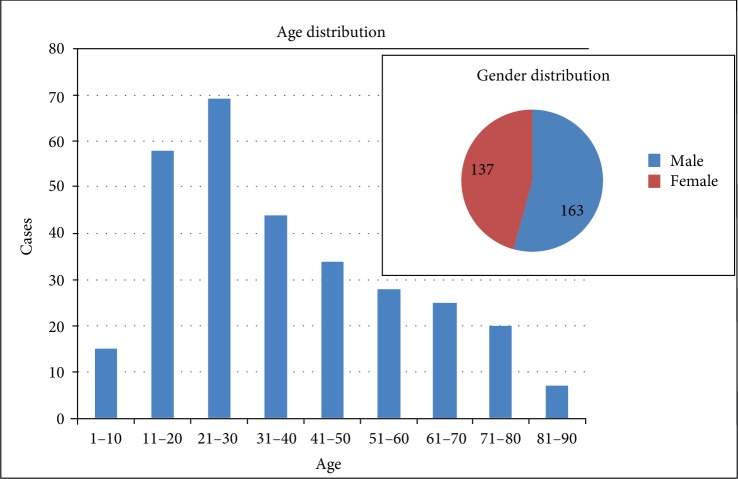
Age and gender distribution of 300 cases of benign tumors and tumor-like lesions.

Between January 2016 and January 2019, 300 pathologically proven (after surgical operation or biopsy) benign bone tumors and tumor-like diseases with integrated MR data from our hospital were retrospectively analyzed. Informed consent was obtained from all participants included in the study. Borderline tumors such as giant-cell tumor of bone and desmoplastic fibroma, lesions with pathological fractures, and cranial lesions were excluded from the study. A total of 300 patients were included in the study, and the age and gender distribution of participants are shown in Figure 1.

### 2.2. Examination

All patients underwent MR plain scan within 10 days before the surgical procedure or other treatments. Enhanced MRI was performed in 50 cases. Follow-up MRI was performed 2 weeks to 3 months after treatment.

**Figure 2:**
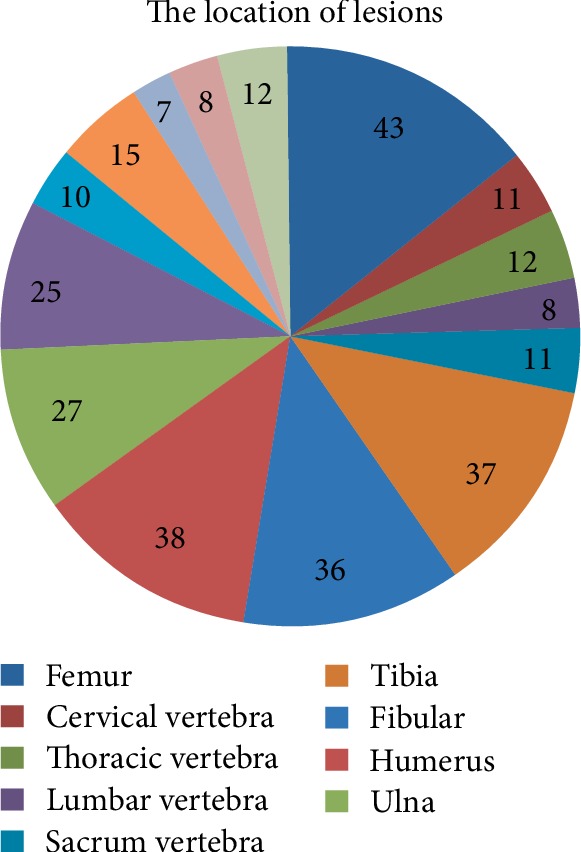
The location of lesions in 300 cases.

The location of lesions is shown in Figure 2.

MRI examination was performed using the GE Signa HDXT 3.0T MR scanner. The MR scan sequence and parameters are shown in Table 1.

### 2.3. Image Analysis

Two senior musculoskeletal radiologists reviewed all images on the PACS system, and differences in opinion were resolved by consensus. The presence of bone marrow and soft tissue edema was first determined using MR images. The types of tumor and tumor-like diseases associated with bone marrow and soft tissue edema and the numbers of preoperative misdiagnosis were determined using the pathology results. Post-operative changes in the incidence of edema were assessed.

### 2.4. Statistical Analysis

The occurrence rates of surrounding edema in different kinds of benign bone tumor and tumor-like diseases were compared. Statistical analyses were performed using the Chi-square test and SPSS software (version 20.0, IBM Corporation, Armonk, NY). All *P* values were two-sided, and a *P*‐value <0.05 was considered statistically significant.

## 3. Results

### 3.1. Pathological Diagnosis

**Figure 3:**
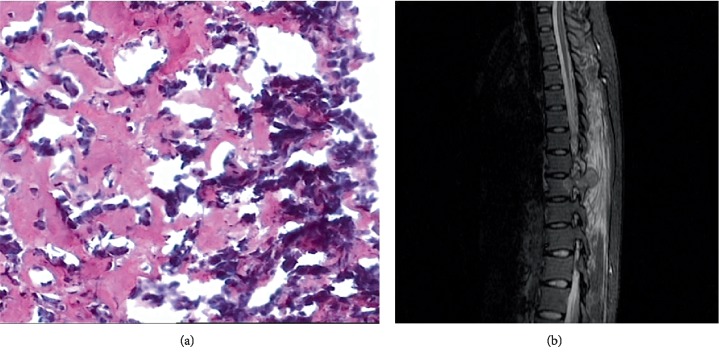
(a) A 48-year-old man with 8th thoracic vertebra osteoblastoma. The pathological slice (HE × 200) shows that the surface of trabeculae constructed by osteoid tissue or woven bone is covered by osteoblastic cells. (b) Preoperative MR T2WI with fat suppression showing the lesion and surrounding soft tissue edema.

**Figure 4:**
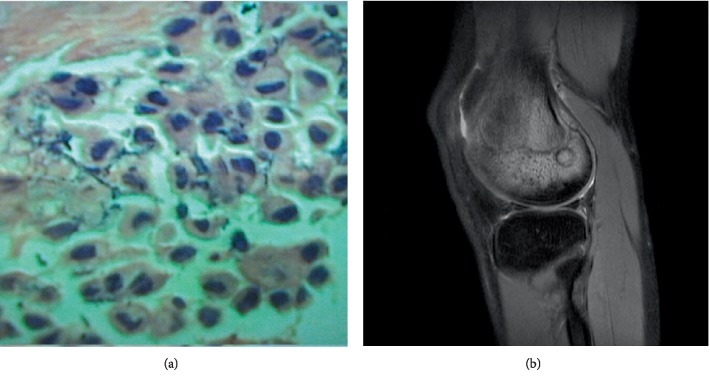
(a) A 32-year-old woman with femur chondroblastoma. The pathological slice (HE × 200) shows that chondroblastic cells are round, polygonal, the margin of the cytoplasm is clear, the nucleus is round or oval, a groove and dent of the nucleus is observed, and few small nucleoli are observed. (b) MR T2WI with fat suppression showing the lesion in the distal femur and surrounding marrow edema.

**Figure 5:**
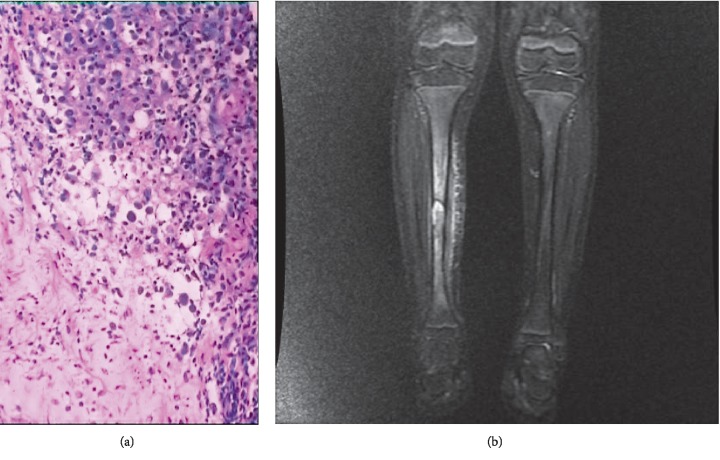
(a) An 8-year-old boy with right tibia Langerhans cell histiocytosis. The pathological slice (HEߙ×ߙ200) shows that cells are oval, the nucleus is folded, the chromatin is fine and smooth, the nucleolus is not obvious, the cytoplasm is moderate, slightly eosinophilic, with the background showing the eosinophilic granulocyte, histocyte, multinucleated giant cells, neutrophile granulocytes, and small lymphocytes (HE × 200). (b) MR T2WI with fat suppression showing bone marrow and soft tissue edema around the obvious bone destruction of the right tibia.

The 300 cases of benign bone tumors and tumor-like diseases included osteoid osteoma, osteoblastoma (Figure 3(a)), chondroblastoma (Figure 4(a)), Langerhans cell histiocytosis (Figure 5(a)), nonossifying fibroma, osteochondroma, fibrous dysplasia of bone, chondroma, simple bone cyst, osteoma, chondromyxoid fibroma, aneurysmal bone cyst, ganglion cyst of bone, capillary hemangioma of bone, and epidermoid cyst of bone. The pathology results and the number of cases of surrounding edema are shown in Table 2.

### 3.2. MRI Findings

Occurrence rate of surrounding edema: Surrounding edema was detected in 107 cases and the occurrence rate was 35.7% (107/300), among which eight cases (7.5%, 8/107) were only surrounding bone marrow edema (Figure 4(b)), six cases (5.6%, 6/107) were only surrounding soft tissue edema (Figure 3(b)), and 93 cases (86.9%, 93/107) were surrounding bone marrow edema together with soft tissue edema (Figure 5(b)).

Occurrence rate of surrounding edema in different benign tumors and tumor-like diseases: Surrounding edema was detected in 27 cases of Langerhans cell histiocytosis (84.4%, 27/32), 19 cases of osteoblastoma (86.4%, 19/22), 31 cases of osteoid osteoma (93.9%, 31/33), and 23 cases of chondroblastoma (85.2%, 23/27). There was no statistically significant difference in the occurrence rate of edema among these diseases (*χ*^2^ = 1.7, *P* > 0.05). Surrounding edema was observed in one case of osteochondroma, one case of chondromyxoid fibroma, two cases of aneurysmal bone cyst, and three cases of capillary hemangioma of bone. No surrounding edema was found in other kinds of benign bone tumors and tumor-like diseases.

Contrast-enhanced MRI was performed in 50 cases, of which 38 were associated with surrounding edema. Among the 38 cases enhanced by contrast agent injection, the degree of enhancement of the edema was higher than that of the lesion in 12 cases, lower than that of the lesion in 13 cases, and comparable to that of the lesion in 13 cases (Table 3).

### 3.3. Misdiagnosis Rate

Forty-nine cases were misdiagnosed as malignant tumors before surgery. The misdiagnosis rate was 45.8% (49/107).

### 3.4. Follow-Up and Changes in the Surrounding Edema after Treatment

**Figure 6:**
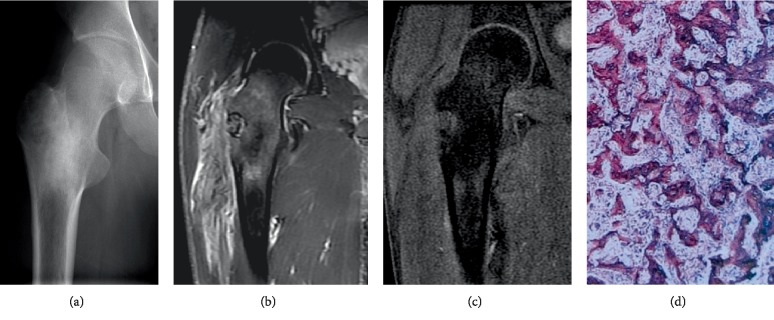
(a)–(d) A 23-year-old boy with femur osteoid osteoma. (a) Preoperative radiograph showing osteosclerosis in the intertrochanter of the femur. (b) At 1 month after the first surgical operation, the bone marrow and soft tissue edema were still observed by MR T2WI with fat suppression. (c) At 2 weeks after the second surgical operation, MR T2WI with fat suppression demonstrated that the bone marrow and soft tissue edema disappeared. (d) The pathological slice of the specimen after second operation showed that the loose fibro-vascular mesenchyma between the trabeculae consisted of osteoid tissue or woven bone (HE × 200).

Among 107 cases with surrounding edema, 89 underwent surgery; 19 cases were followed-up by MR during a period of 2 weeks to 3 months postoperatively. The surrounding edema disappeared after surgery in 17 cases, including three cases of Langerhans cell histiocytosis, four cases of osteoblastoma, six cases of osteoid osteoma, and four cases of chondroblastoma. The surrounding edema was still detected in two cases of osteoid osteoma at 1 month after the first surgical operation; post-operative CT scan showed the persistence of the tumor nest, suggesting that the tumor nests were not excised or were incompletely excised. After resection of the tumor nests in second surgical operations, the surrounding edema disappeared (Figures 6(a)–6(c)). The pathology of the specimen from the second operation showed fibro-vascular mesenchyma between trabeculae constructed by osteoid tissue or woven bone (Figure 6(d)).

Four cases of Langerhans cell histiocytosis underwent chemotherapy; the range of surrounding edema was reduced after 2 weeks. Two cases underwent radiotherapy, and the range of edema was reduced after 2 weeks.

Twelve cases did not receive treatment; there was no significant change in the surrounding edema after 2 weeks.

## 4. Discussion

### 4.1. Occurrence Rate of Surrounding Edema in Benign Bone Tumors and Tumor-Like Diseases

The results of the study by Kroon et al. [11] showed that surrounding bone marrow edema and soft tissue edema are detected in 42% (10/24) and 58% (14/24) of benign bone tumors and tumor-like diseases, respectively, which was a higher rate than that of the present study at 35.7% (107/300). One possible explanation for this difference is that the patient population was small (24 cases) and there were few disease types (7 kinds) included in the study by Kroon et al. [11]. The occurrence of surrounding edema is closely related to the numbers of patients and disease types. In the present study, surrounding edema was not detected in nonossifying fibroma, fibrous dysplasia of bone, chondroma, simple bone cyst, osteoma, and ganglion cyst of bone. The inclusion of additional cases of these diseases may have decreased the occurrence rate of surrounding edema. Regardless of the higher or lower occurrence rate of edema, the above results indicate that surrounding edema was not a specific indicator of malignant bone tumors.

### 4.2. Surrounding Edema in Different Types of Benign Bone Tumor and Tumor-Like Diseases

The occurrence rates of surrounding edema are relatively high in certain kinds of benign bone tumors and tumor-like diseases. In the present study, the occurrence rate of surrounding edema was 84.4% (27/32) in Langerhans cell histiocytosis, which was slightly lower than that of 91% (20/22) reported by Jeh et al. [12]. The occurrence rate of surrounding edema in osteoblastoma was 86.4% (19/22), which was comparable to that of other studies. Kroon et al. [13] reported that the three cases of osteoblastoma analyzed were associated with surrounding edema. Shaikh et al. [14] reported that 10 of 11 cases of vertebral osteoblastoma were accompanied by surrounding edema. The occurrence rate of surrounding edema in osteoid osteoma in the present study was 93.9% (31/33), which was higher than that of 88.37% (38/43) reported by Davies et al. [15]. Surrounding edema was found in 85.2% (23/27) of chondroblastoma cases in the present study, which was comparable to the rate of 77–92% reported previously [16, 17]. Although the three cases of capillary hemangioma of bone included in the present study were associated with surrounding edema, the sample was too small, and the results need to be confirmed in a greater number of cases. Surrounding edema was found in 18.2% (2/11) of aneurysmal bone cysts in our study, which was a lower rate than that of 33% reported by Woertler et al. [18]. Because aneurysmal bone cysts are often accompanied by pathological fracture, whether the surrounding edema is caused by the pathological fracture or the expanding cortex in cases of aneurysmal bone cyst needs to be determined.

### 4.3. The Performance of Contrast-Enhanced MRI

Among 50 cases that underwent contrast-enhanced MRI, 38 had surrounding edema, and all cases showed enhancement. No new surrounding edema was observed in the enhanced images, and there was no significant difference in the incidence of surrounding edema between the plain scan and the enhanced scan. These results were consistent with those reported by Giraudo et al. [19]. The boundaries of surrounding edema and lesions are clearer in contrast enhanced than in non-contrast enhanced images, as shown by Liu et al. [20], who reported that enhanced MRI imaging can depict osteoid osteomas with greater accuracy than non-enhanced MRI, facilitating the detection of the size and morphology of the lesions.

### 4.4. Limitations of the Study

The present study had several limitations. Firstly, we did not examine the relationship between tumor size and surrounding edema or the relationship between the size of edema and the properties of the lesions. Secondly, although contrast-enhanced MRI was included, dynamic contrast-enhanced MRI was not performed. Thirdly, bone marrow and soft tissue edema were not examined separately because they were detected together in most cases (86.9%, 93/107). In addition, the disease types included in the present study were limited, and the inclusion of a larger cohort is needed. Foti et al. [21] reported that dual-energy CT represents an accurate imaging tool for bone marrow edema of the ankle compared with MRI. We did not perform a comparative study of MRI and dual-energy CT.

## 5. Conclusion

Marrow and soft tissue edema are a common occurrence associated with benign bone tumors and tumor-like diseases. There was no statistically significant difference in the incidence of edema between Langerhans cell histiocytosis, osteoblastoma, osteoid osteoma, and chondroblastoma.

## Figures and Tables

**Table 1:** MR scan sequence and parameters.

Sequence	TR/TE	Slice thickness	Dose
SE-T1WI	500/20	4 mm	
T2WI	4000/80	4 mm	
FS T2WI/STIR	4000/80	4 mm	
T1-FS post-contrast	500/20	4 mm	0.2 ml/kg

**Table 2:** Pathology of 300 cases and number of cases with surrounding edema.

Benign tumors and tumor like diseases	Cases	Edema
Osteoid-osteoma	33	31
Osteoblastoma	22	19
Chondroblastoma	27	23
Langerhans cell histiocytosis	32	27
Nonossifying fibroma	31	0
Osteochondroma	24	1
Fibrous dysplasia of bone	23	0
Chondroma	24	0
Simple bone cyst	20	0
Osteoma	21	0
Chondromyxoid fibroma	12	1
Aneurysmal bone cyst	11	2
Ganglion cyst of bone	10	0
Capillary hemangioma of bone	3	3
Epidermoid cyst of bone	7	0

**Table 3:** Fifty cases of benign tumors and tumor-like diseases with enhanced MRI.

Benign tumors and tumor like diseases	Cases	Edema	Enhancement	
Osteoid-osteoma	7	7		7
Osteoblastoma	6	6		6
Chondroblastoma	11	11		11
Langerhans cell histiocytosis	12	12		12
Nonossifying fibroma	3	0		0
Osteochondroma	4	1		1
Chondromyxoid fibroma	2	0		0
Aneurysmal bone cyst	4	0		0
Capillary hemangioma of bone	1	1		1

## Data Availability

The data used to support the findings of this study are available from the corresponding upon request.
